# Visceral Adiposity Index Is Associated With the Development of Increased Urinary Albumin Excretion and Chronic Kidney Disease: A Population-Based Study

**DOI:** 10.1155/ije/1173231

**Published:** 2025-03-27

**Authors:** Junyan Yu, Chulin Huang, Jiabin Lin, Diaozhu Lin, Feng Li, Yiqin Qi, Ying Liang, Chuan Wang, Leiqin Cai, Wanting Feng, Na Li, Guojuan Lao, Huisheng Xiao, Chuan Yang, Li Yan, Meng Ren, Kan Sun

**Affiliations:** Department of Endocrinology, Sun Yat-sen Memorial Hospital, Sun Yat-sen University, 107 Yanjiang West Road, Guangzhou 510120, China

**Keywords:** albuminuria, chronic kidney disease, cohort study, visceral adiposity index, visceral fat

## Abstract

**Background:** In recent years, the impact of visceral fat accumulation on renal damage has garnered significant attention. However, whether visceral fat accumulation contributes to the incidence of both albuminuria and chronic kidney disease (CKD) is still uncertain. Our objective is to look into the possible correlation between visceral adiposity accumulation and incident increased urinary albumin excretion and CKD.

**Methods:** We analyzed data from a cohort of 9916 subjects aged 40 years and above. As an innovative and convenient biomarker of visceral adiposity distribution, visceral adiposity index (VAI) was calculated in accordance with a gender-specific equation using measurement of blood lipids and anthropometric parameters of obesity. Albuminuria was determined by urine albumin-to-creatinine ratio (UACR) ≥ 30 mg/g. CKD was determined by establishment of either of the following: (1) estimated glomerular filtration rate (eGFR) 60 mL/min per 1.73 m^2^ or less; (2) UACR ≥ 30 mg/g.

**Results:** During an average follow-up period of 3.6 ± 0.7 years, 245 (4.7%) subjects developed albuminuria and 332 (6.3%) participants developed CKD. Incidence of albuminuria and CKD had a tendency to advance along with ascending VAI levels in both genders. According to multiple stepwise linear regression analysis, γ-glutamyltransferase (γ-GGT), fasting insulin, fasting plasma glucose (FPG), low-density lipoprotein cholesterol (LDL-C), and systolic blood pressure (SBP) were independent determinants for VAI. Multivariate-adjusted hazard ratios (HRs) of albuminuria with 95% confidence intervals (CIs) in Cox regression analysis were 1 (reference), 0.82 (0.53–1.29), 1.50 (1.01–2.23), and 1.52 (1.02–2.26) in ascending quartiles of VAI. Similarly, the HRs with 95% CI of CKD in ascending quartiles of VAI were 1 (reference), 0.96 (0.66–1.41), 1.51 (1.07–2.15), and 1.56 (1.10–2.20). For subgroup analyses, VAI significantly correlated with risk of both albuminuria and CKD in older subjects (age ≥ 58 years), nondiabetes subjects, and non-ASCVD subjects (all *p* < 0.05).

**Conclusions:** The greater deposition of visceral fat assessed by VAI is independently associated with risk of increased urinary albumin excretion and CKD in middle-aged and aged Chinese.

## 1. Introduction

Globally, chronic kidney disease (CKD) was demonstrated as a leading cause to mortality and decreasing disability-adjusted life years [1, 2]. There is a high incidence of increased urinary albumin excretion among the general population and also in people suffering from specific diseases like diabetes or hypertension [3]. In addition to being a reliable predictor of renal insufficiency, albuminuria also shows association with a higher prevalence of angiocardiopathy and mortality for all causes [4–8]. Even after adjusting for the presence of diabetes and cardiovascular disorders, albuminuria is still an effective indicator to determine the likelihood of adverse outcomes, such as chronic heart failure–related death or hospitalization [9]. Therefore, identifying early risk factors for albuminuria and CKD has become increasingly essential.

It is well recognized that the distribution of body fat is a significant factor in predicting adverse metabolic outcomes independently of total body fat [10]. Previous studies suggest that visceral fat and kidney damage are more closely related than subcutaneous adiposity [11–14]. However, despite visceral adiposity being identified accurately by computed tomography (CT) and magnetic resonance imaging (MRI), these measurements are not practical in light of their high cost and radiant exposure [15]. Visceral adiposity index (VAI), which is calculated according to body mass index (BMI), waist circumference (WC), triglyceride (TG), and high-density lipoprotein cholesterol (HDL-C), has been proposed as a reliable index of CT-measured visceral adiposity area [16].

Based on our previous clinical investigation, a significant association was found between VAI and the prevalence of albuminuria [17]. Nevertheless, there was a lack of a long-term cohort study to assess the association of VAI with incident albuminuria and CKD. A longitudinal cohort study on 2809 participants from the Korean population reported that both a higher baseline level and a more substantial increase in visceral fat mass were considered important risk factors for the onset of proteinuria [18]. However, they evaluated albuminuria by dipstick urinalysis, which had much lower sensitivity for detecting renal dysfunction than urine albumin-to-creatinine ratio (UACR) [19]. Therefore, we explored the possible association of VAI with risk of increased urinary albumin excretion and CKD in the present study.

## 2. Methods

### 2.1. Study Population and Design

**Figure 1:**
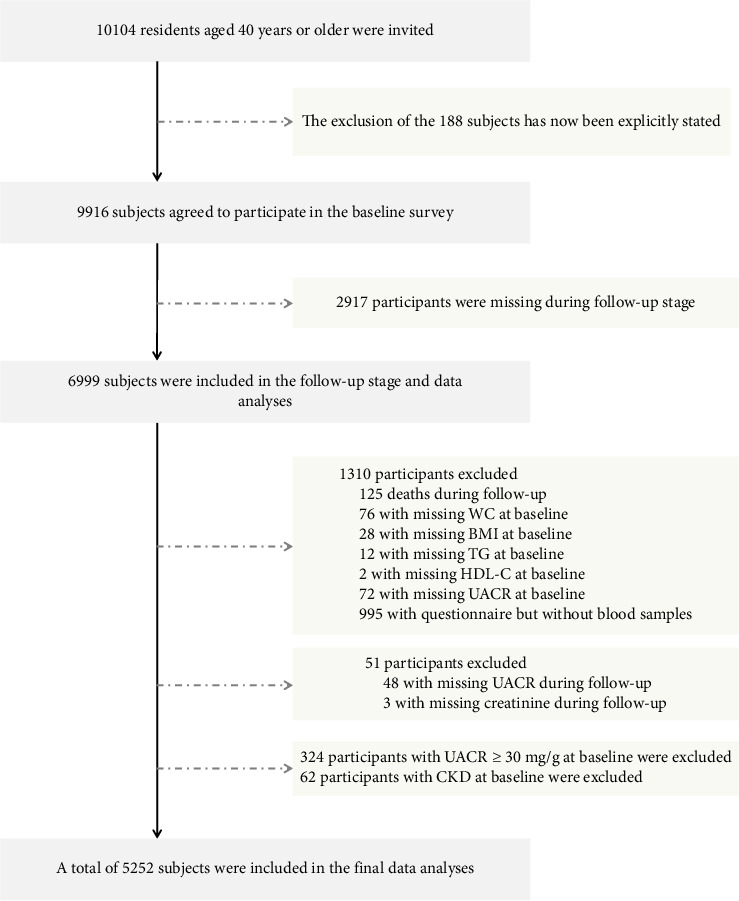
Flowchart of the population selection of the present study.

We conducted a cohort study among residents of Guangzhou communities, with recruitment carried out from June to November 2011. The follow-up was completed in a single visit between 2014 and 2016. The study population was derived from the Risk Evaluation of cAncers in Chinese diabeTic Individuals: a lONgitudinal (REACTION) Study, a multicenter, prospective observational study aimed at assessing chronic diseases within the Chinese population [20, 21]. Before collecting information, we obtained informed consent from all participants by having them sign an informed consent form. All registered permanent residents were invited to participate in the screening examinations through poster advertisements, telephone calls, or home visits. The study design and protocol for this cohort have been previously described in detail [17]. As shown in Figure 1, a total of 10,104 subjects aged 40 and above were invited to participate in this project. Of these, 9916 individuals agreed to endorse the document of consent and participate in the baseline survey. A total of 188 subjects who did not agree to participate in the baseline investigation were excluded from the analyses. Subsequently, we excluded 2917 participants who were missing during follow-up stage or met the following conditions: failure to provide baseline information (WC: *n* = 76; BMI: *n* = 28; TG, *n* = 12; HDL-C, *n* = 2; UACR, *n* = 72), finishing the questionnaire without blood sample (*n* = 995), deaths during follow-up (*n* = 125), failure to provide information during follow-up (UACR, *n* = 48; creatinine, *n* = 3), UACR ≥ 30 mg/g at baseline (*n* = 324), and confirmed CKD at baseline (*n* = 62). In total, 5252 patients were eligible for final data analysis. The research protocol for this study received approval from the Institutional Review Board (IRB) of Sun Yat-sen Memorial Hospital affiliated with Sun Yat-sen University, in accordance with the principles outlined in the Helsinki Declaration II.

### 2.2. Data Collection

We collected sociodemographic, family history, and lifestyle information of each subject, via standard questionnaires administered by trained staff members. This study classified smoking or drinking habits according to whether they were “never,” “ever” (no smoking, drinking over 6 months), or “current” (smoking or drinking regularly in the past 6 months) [22]. Leisure-time physical activity was assessed using a simplified version of the International Physical Activity Questionnaire (IPAQ), which included questions regarding exercise intensity, frequency, and duration [23]. We evaluated metabolic equivalent hours per week (MET-h/week) as a means of assessing overall physical activity. Participants were instructed to follow standardized procedures to measure their blood pressure, height, and weight with the aid of skilled staff [20]. Blood pressure was determined by the mean of three consecutive measurements. During the current study, a BMI of 28.0 or greater was considered obesity, while a BMI of 24.0 to 27.9 was considered overweight [24]. Arteriosclerotic cardiovascular disease (ASCVD) was defined as the occurrence of nonfatal acute myocardial infarction or coronary heart disease death or fatal or nonfatal stroke [25].

During the morning hours of 7:00 a.m. to 9:00 a.m., individual blood samples were obtained after a minimum overnight fast of 10 h. Using an autoanalyzer (Beckman CX-7 Biochemical Autoanalyzer, Brea, CA, USA), the following laboratory tests were completed: fasting plasma glucose (FPG), fasting serum insulin, γ-glutamyltransferase (γ-GGT), aspartate aminotransferase (AST), alanine aminotransferase (ALT), low-density lipoprotein cholesterol (LDL-C), and total cholesterol (TC). The Modification of Diet in Renal Disease (MDRD) equation was applied for calculating the estimated glomerular filtration rate (eGFR) expressed in mL/min per 1.73 m^2^ via a formula of eGFR = 186 × [serum creatinine × 0.011]^−1.154^ × [age]^−0.203^ × [0.742 if female] × 1.233, in which serum creatinine levels were measured in μmol/L, and 1.233 was the modifying coefficient for Chinese population [26]. Following the 2010 diagnostic criteria of the American Diabetes Association (ADA) [27], the diagnosis of diabetes was established when any of the following criterions were present: FPG ≥ 7.0 mmol/L and/or the 2 h oral glucose tolerance test (OGTT) plasma glucose level ≥ 11.1 mmol/L and/or hemoglobin A1c (HbA1c) ≥ 6.5%.

### 2.3. Definitions of VAI, Albuminuria, and CKD

The following formulas were used to determine the VAI using gender-specific equations: men: [WC/(39.68+(1.88 × BMI))] × (TG/1.03) × (1.31/HDLC); women: [WC/(36.58+(1.89 × BMI))] × (TG/0.81) × (1.52/HDLC) [28]. In these formulas, WC was measured in cm, and HDL-C and TG were measured in mmol/L. Urinalysis was conducted using first-morning spot urine samples. Utilizing chemiluminescence immunoassay (Siemens Immulite 2000, USA) and a modified Jaffe method (Biobase-Crystal, Jinan, China) on the automatic analyzer, the concentrations of urine albumin and creatinine were assessed. UACR, expressed in mg/g, was determined by dividing the urinary albumin concentrations by the urinary creatinine concentrations. Albuminuria was determined by UACR ≥ 30 mg/g. CKD was determined by establishment of either of the following: (1) eGFR 60 mL/min per 1.73 m^2^ or less; (2) UACR ≥ 30 mg/g.

### 2.4. Statistical Analysis

It should be noted that we included data from a single follow-up in the analysis. The purpose of our study design was to observe the association between VAI measured in 2011 and the incidence of kidney dysfunction in the subsequent years through long-term follow-up. Continuous variables were presented as means ± standard deviations (SDs) for normally distributed data and medians (interquartile ranges) for non-normally distributed data. All categorical parameters were expressed as numbers (proportions). Skewed distribution variables such as UACR, MET-h/week, and TG were logarithmically transformed before analysis. To assess the trends of the characteristics of demographics and clinical condition across groups divided by VAI quartiles, linear regression analysis was implemented. Comparisons of clinical features as well as laboratory indicators were conducted among groups utilizing one-way ANOVA, whereas post hoc comparisons were made utilizing the Bonferroni correction. The comparisons between categorical variables were performed adopting the *χ*2 test.

In order to determine the factors independently correlated to VAI, potential covariates that showed significance at *p* < 0.20 in Pearson's correlations were applied to the multivariate stepwise linear regression models. Meanwhile, we assessed the associations between VAI level and the risks of albuminuria and CKD using Cox regression analysis, considering both unadjusted and multivariate-adjusted models. Adjusted variables identified as potential covariates or significant in the stepwise linear regression were included in the multivariate-adjusted Cox regression analysis. Model 1 represented the model without adjustments. Model 2 included an adjustment for age. Model 3 included adjustments for age, sex, physical activity, and systolic blood pressure (SBP). Model 4 included adjustments for age, sex, physical activity, SBP, LDL-C, FPG, fasting insulin, and γ-GGT. The hazard ratios (HRs) along with their corresponding 95% confidence interval (CI) were determined. Next, we performed subgroup analyses to examine the relationship between VAI values and the incidence of albuminuria and CKD, with the use of stratified variables including median age (≥ 58 and < 58 years), gender (men and women), body size (normal, overweight, and obesity), and the establishments of central obesity, diabetes, hypertension, and ASCVD (yes/no). In the interaction analyses, we examined whether these strata factors had potential modifiers that could alter the correlation between VAI and incidence of albuminuria and CKD. Tests for interaction were conducted, incorporating each subgroup–strata factor, quartiles of VAI level, and their respective interaction terms (strata variable multiplied by quartiles of VAI level) into the models.

Two-sided statistical tests were used, and statistical significance was determined with a *p* value threshold of < 0.05. The statistical analysis was carried out with the use of SAS Version 9.3 (SAS Institute Inc., Cary, NC, USA).

## 3. Results

Among the 5252 eligible individuals, the mean age of the cohort was 55.7 ± 7.1 years. During an average follow-up period of 3.6 ± 0.7 years, 245 (4.7%) subjects developed albuminuria and 332 (6.3%) participants developed CKD. Clinical and biochemistry-based characteristics of the study population at follow-up are presented in Table 1, according to baseline VAI quartiles. Baseline VAI level was positively associated with follow-up measured UACR, age, BMI, WC, SBP, diastolic blood pressure (DBP), incidence of previous hypertension, TG, TC, FPG, fasting insulin, ALT, and γ-GGT, while it was negatively associated with eGFR, proportion of males, and HDL-C level (all *p* for trend < 0.0001).

**Figure 2:**
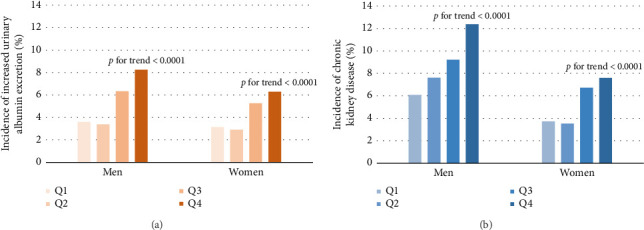
Incidence of increased urinary albumin excretion and CKD in different quartiles of visceral adiposity index levels.

As shown in Table 2, Pearson's correlation revealed that VAI level was significantly correlated with age, SBP, DBP, physical activity level, LDL-C, FPG, fasting insulin, ALT, γ-GGT, UACR, and eGFR. Further multivariate stepwise linear regression showed that SBP, LDL-C, FPG, fasting insulin, and γ-GGT were independent determinants for VAI. From the lowest quartile to the highest quartile of VAI, the incidence of albuminuria ranged from 3.6%, 3.38%, 6.34%, and 8.25% in men and 3.14%, 2.91%, 5.27%, and 6.29% in women, respectively. In a similar manner, the incidence of CKD was 6.08%, 7.61%, 9.22%, and 12.38% in men and 3.72%, 3.53%, 6.72%, and 7.59% in women (Figure 2, all *p* for trend < 0.0001). The univariate Cox regression analysis (Model 1) showed that subjects with VAI in the third and fourth quartiles were more likely to develop albuminuria and CKD compared to those in the first quartile (Table 3). After adjusting for age, sex, physical activity, SBP, LDL-C, FPG, fasting insulin, and γ-GGT (Model 4), the HRs of albuminuria for increasing VAI quartiles were 1 (reference), 0.82 (0.53–1.29), 1.50 (1.01–2.23), and 1.52 (1.02–2.26) (*p* for trend = 0.0029). Besides, after further adjustment in Model 4, the HRs (95% CI) of CKD were 1 (reference), 0.96 (0.66–1.41), 1.51 (1.07–2.15), and 1.56 (1.10–2.20) in ascending VAI quartiles. Additionally, the HRs (95% CI) of albuminuria and CKD for one quartile increase of VAI in Model 4 were 1.21 (1.06–1.37) and 1.20 (1.07–1.33) after adjusting for multiple confounders, respectively.

**Figure 3:**
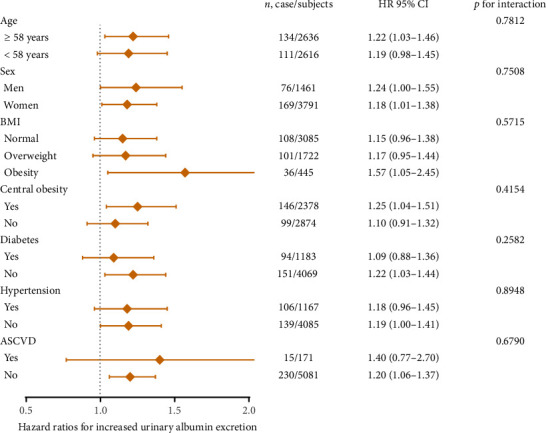
Risk of incident increased urinary albumin excretion with each quartile increase of visceral adiposity index in different subgroups.

**Figure 4:**
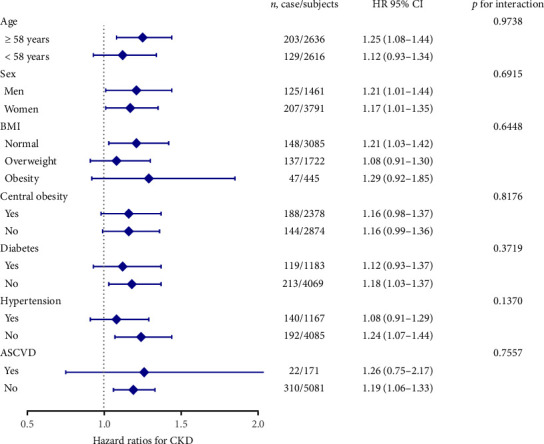
Risk of incident CKD with each quartile increase of visceral adiposity index in different subgroups.

For subgroup analyses, forest plots revealed that VAI level was not consistently correlated with albuminuria and CKD among different subgroups (Figures 3 and 4). VAI significantly correlated with risk of albuminuria in older subjects (age ≥ 58 years), women, obese subjects, centrally obese subjects, nondiabetes subjects, and non-ASCVD subjects (all *p* < 0.05). Meanwhile, VAI also showed a significant association with CKD among older subjects (age ≥ 58 years), both genders (men and women), subjects with normal BMI, nondiabetes subjects, nonhypertension subjects, and non-ASCVD subjects (all *p* < 0.05). No statistically significant interaction was observed between VAI quartiles and each component within the strata.

## 4. Discussion

In this longitudinal cohort study of middle-aged and elderly individuals from China, the incidence of albuminuria as well as CKD was significantly associated with visceral fat accumulation evaluated by VAI. Unlike anthropometric indices (BMI and WC) and commonly used lipid metabolism indicators (HDL and TG), VAI has proved to be a superior and reliable indicator of visceral adiposity. An important strength of VAI is that it requires less intensive and low-cost measurements compared to MRI and CT. Given this clear benefit, clinical applications of VAI could lead to a cost-effective and convenient means of screening for kidney dysfunction at an early stage.

Previous research has indicated that VAI is presumably associated with kidney dysfunction. By including 23,570 participants aged over 18, Chen et al. [29] found that elevated VAI levels appeared to correlate with an enhanced prevalence of CKD in a cross-sectional study. In contrast, identifying early warning indicators of albuminuria was also crucial [30]. Nevertheless, in that study, renal dysfunction was only assessed by eGFR levels without evaluation of albuminuria. Moreover, it should be noted that their results only applied to relatively healthy adults. The research sample in their study consisted of selected individuals who were both youth and middle-aged, reasonably healthy, and most of whom free from any major comorbid conditions or diseases. Besides, a study conducted by Wang et al. [31] examined 24,871 individuals with prediabetes and observed a positive association between VAI and UACR among prediabetic Chinese. The variables in the studies mentioned above were assessed at a single time point, and the absence of longitudinal follow-up prevented the establishment of a causal relationship between VAI and albuminuria. Currently, we conducted the largest retrospective longitudinal cohort investigation exploring the relationships of VAI with incident albuminuria and CKD. In our investigation, we analyzed 5252 Chinese individuals aged 40 and above, and our findings indicated that increased visceral fat deposition, as determined by VAI, was correlated with elevated occurrences of albuminuria and CKD, after accounting for diverse demographic and clinical factors. In this context, VAI has the capability to act as a predictive indicator of both albuminuria and CKD. Consequently, when devising clinical strategies for intervening in early-stage renal dysfunction, enhanced attention should be paid to this particular aspect.

The biological mechanisms that explain the association between visceral fat deposition and renal damage have not yet been fully understood. In the process of visceral adipose tissue remodeling, visceral adipocytes secrete increased free fatty acids (FFAs), which accumulate in skeletal muscle and liver [32]. It has been reported that while obese adipose tissue experiences remodeling and expansion to accommodate a high intake of energy, adipokine secretion undergoes a significant change. For example, adiponectin secretion declines along with excess accumulation of visceral adiposity, while leptin and proinflammatory adipokines are secreted in increasing amounts [33, 34]. Increased FFA level and derangements of adipokines are involved with systemic inflammation, insulin resistance, and hypertension [35]. The above pathologic changes contribute to hyperfiltration-induced renal injury including glomerulomegaly, proteinuria, podocyte deletion, focal segmental glomerulosclerosis, and interstitial fibrosis [36, 37]. Moreover, adipocyte hypertrophy stimulates the release of very-low-density lipoproteins (VLDLs) as well as TG, both of which are atherogenic and may result in renal dysfunction [38]. In addition, visceral adipocytes secrete angiotensin, which not only arouses the activation of renin–angiotensin system but also induces hypertension, glomerular hyperfiltration, and renal injury [39, 40]. Despite this, there is a need for further clarification and understanding of the pathophysiological mechanisms involved in the processes mentioned above.

The study has certain limitations that need to be considered. Firstly, in light of the concern that 3.6 years of follow-up may not be sufficient to demonstrate an accurate association between VAI and kidney damage, we acknowledge that the duration of follow-up may not capture the full progression of kidney dysfunction. Further studies with multiple follow-up visits over longer periods are anticipated to collect more outcome events, which would provide more stable and reliable conclusions. Secondly, while VAI offers a convenient and practical approach to evaluate visceral fat dysfunction, the utilization of advanced imaging techniques such as MRI remains widely recognized as the gold standard for assessing visceral adiposity distribution. However, large-scale epidemiological studies have been hindered from adopting this method due to constraints related to research funding, site logistics, and participant compliance considerations. Thirdly, after undergoing a physical examination at the study's onset, people may adjust their lifestyle and dietary practices, which could consequently lead to alterations in VAI. Nonetheless, our investigation only encompassed recording the baseline and follow-up data pertaining to VAI, without delving into the relationship between fluctuations in VAI and the incidence of albuminuria and CKD.

## 5. Conclusions

In summary, among middle-aged and elderly individuals from China, our findings suggest that the accumulation of visceral fat, as measured by VAI, is independently associated with the occurrence of increased urinary albumin excretion and CKD.

## Figures and Tables

**Table 1:** Characteristics of study population at follow-up by baseline visceral adiposity index quartiles.

	Quartile 1	Quartile 2	Quartile 3	Quartile 4	*p* for trend
*n* (%)	1305 (24.9)	1317 (25.1)	1314 (25.0)	1316 (25.1)	
VAI	0.83 (0.68–0.96)	1.33 (1.19–1.47)⁣^∗^	2.00 (1.80–2.30)⁣^∗^^#^	3.71 (3.06–5.16)⁣^∗^^#&^	< 0.0001
UACR (mg/g)	6.72 (5.10–9.67)	7.22 (5.52–10.23)⁣^∗^	7.43 (5.60–11.16)⁣^∗^	8.17 (5.77–12.45)⁣^∗^^#&^	< 0.0001
eGFR (ml/min per 1.73 m^2^)	91.1 ± 13.7	89.7 ± 13.6	89.5 ± 14.0	87.7 ± 13.4⁣^∗^^#&^	< 0.0001
Age (years)	58.6 ± 7.3	59.1 ± 7.1⁣^∗^	59.4 ± 6.9⁣^∗^	60.0 ± 7.2⁣^∗^^#^	< 0.0001
Male [*n* (%)]	444 (34.0)	355 (27.0)	347 (26.4)	315 (23.9)	< 0.0001
BMI (kg/m^2^)	22.3 ± 3.2	23.2 ± 3.1⁣^∗^	24.0 ± 3.4⁣^∗^^#^	24.6 ± 3.6⁣^∗^^#&^	< 0.0001
WC (cm)	79.7 ± 8.8	82.6 ± 9.0⁣^∗^	85.0 ± 9.5⁣^∗^^#^	86.6 ± 8.5⁣^∗^^#&^	< 0.0001
SBP (mmHg)	120.2 ± 15.8	123.0 ± 15.3⁣^∗^	125.5 ± 15.2⁣^∗^^#^	128.3 ± 16.1⁣^∗^^#&^	< 0.0001
DBP (mmHg)	71.0 ± 9.9	72.4 ± 9.3⁣^∗^	73.7 ± 9.6⁣^∗^^#^	75.1 ± 9.6⁣^∗^^#&^	< 0.0001
Current smoking [*n* (%)]	126 (12.7)	125 (12.6)	130 (13.2)	123 (12.7)	0.929
Current drinking [*n* (%)]	72 (7.0)	53 (5.1)	53 (5.2)	55 (5.5)	0.159
Physical activity (MET-h/week)	30.0 (15.0–54.0)	27.0 (12.0–49.0)	30.0 (12.0–50.0)	24.0 (10.5–45.0)⁣^∗^	0.008
Previous hypertension [*n* (%)]	132 (10.1)	160 (12.2)	204 (15.5)	273 (20.7)	< 0.0001
Previous ASCVD [*n* (%)]	29 (2.2)	45 (3.4)	49 (3.7)	48 (3.7)	0.037
TG (mmol/L)	0.94 (0.76–1.20)	1.24 (0.99–1.55)⁣^∗^	1.50 (1.17–1.93)⁣^∗^^#^	2.19 (1.61–3.00)⁣^∗^^#&^	< 0.0001
TC (mmol/L)	5.73 ± 1.04	5.89 ± 1.08⁣^∗^	5.99 ± 1.13⁣^∗^	6.01 ± 1.19⁣^∗^^&^	< 0.0001
HDL-C (mmol/L)	1.65 ± 0.35	1.47 ± 0.29⁣^∗^	1.35 ± 0.28⁣^∗^^#^	1.22 ± 0.24⁣^∗^^#&^	< 0.0001
LDL-C (mmol/L)	3.38 ± 0.87	3.63 ± 0.92⁣^∗^	3.73 ± 0.94⁣^∗^^#^	3.50 ± 0.99⁣^∗^^#&^	0.0001
FPG (mmol/L)	5.10 (4.77–5.55)	5.20 (4.87–5.66)⁣^∗^	5.30 (4.88–5.82)⁣^∗^^#^	5.42 (5.00–6.07)⁣^∗^^#&^	< 0.0001
Fasting insulin (μIU/mL)	5.80 (4.40–7.70)	7.00 (5.20–9.20)⁣^∗^	7.80 (5.80–10.20)⁣^∗^^#^	9.30 (6.90–12.20)⁣^∗^^#&^	< 0.0001
ALT (U/L)	10.0 (7.0–15.0)	11.0 (8.0–16.0)⁣^∗^	12.0 (8.0–17.0)⁣^∗^	13.0 (9.0–19.0)⁣^∗^^#&^	< 0.0001
AST (U/L)	19.0 (16.0–22.0)	18.0 (16.0–22.0)	18.0 (16.0–22.0)	19.0 (16.0–22.5)^#^	0.020
γ-GGT (U/L)	20.0 (15.0–27.0)	21.0 (16.0–30.0)⁣^∗^	23.0 (18.0–33.0)⁣^∗^^#^	27.0 (20.0–38.0)⁣^∗^^#&^	< 0.0001

*Note:* Data were presented as means ± SD or medians (interquartile ranges) for skewed variables or numbers (proportions) for categorical variables. *p* for trend was calculated for the linear regression analysis tests across the groups. The *p* values for trend were calculated using ANOVA for continuous variables and χ^2^ tests for categorical variables. γ-GGT, γ-glutamyltransferase; MET-h/week: separate metabolic equivalent hours per week.

Abbreviations: ASCVD, atherosclerotic cardiovascular disease; BMI, body mass index; DBP, diastolic blood pressure; eGFR, estimated glomerular filtration rate; FPG, fasting plasma glucose; HDL-C, high-density lipoprotein cholesterol; LDL-C, low-density lipoprotein cholesterol; SBP, systolic blood pressure; TC, total cholesterol; TG, triglycerides; UACR, urinary albumin-to-creatinine ratio; VAI, visceral adiposity index; WC, waist circumference.

⁣^∗^*p* < 0.05 compared with Quartile 1 of VAI.

^#^ *p* < 0.05 compared with Quartile 2 of VAI.

^&^ *p* < 0.05 compared with Quartile 3 of VAI.

**Table 2:** Pearson's correlation and stepwise regression analysis of baseline determinants of visceral adiposity index.

	*r*	*p* value	Standardized β	*p* value
Age (years)	0.05	< 0.0001	—	—
SBP (mmHg)	0.21	< 0.0001	0.09	< 0.0001
DBP (mmHg)	0.19	< 0.0001	—	—
Physical activity (MET-h/week)	−0.02	0.045	—	—
LDL-C (mmol/L)	−0.03	0.024	−0.07	< 0.0001
FPG (mmol/L)	0.17	< 0.0001	0.05	0.0002
Fasting insulin (μIU/mL)	0.42	< 0.0001	0.35	< 0.0001
ALT (U/L)	0.13	< 0.0001	—	—
AST (U/L)	0.01	0.564	—	—
γ-GGT (U/L)	0.25	< 0.0001	0.15	< 0.0001
UACR (mg/g)	0.09	< 0.0001	—	—
eGFR (ml/min per 1.73 m^2^)	−0.06	< 0.0001	—	—

*Note: r*, correlation coefficient; standardized β, regression coefficient.

**Table 3:** The risk of increased urinary albumin excretion and CKD according to quartiles of visceral adiposity index.

		Quartile 1	Quartile 2	Quartile 3	Quartile 4	P for trend	One quartile increase of VAI
Increased urinary albumin excretion	Model 1	1	0.91 (0.59–1.42)	1.72 (1.76–2.54)	2.13 (1.47–3.09)	< 0.0001	1.35 (1.20–1.52)
	Model 2	1	0.91 (0.59–1.41)	1.70 (1.15–2.49)	2.05 (1.41–2.98)	< 0.0001	1.33 (1.18–1.50)
	Model 3	1	0.84 (0.54–1.31)	1.56 (1.06–2.30)	1.74 (1.19–2.56)	0.0002	1.27 (1.12–1.43)
	Model 4	1	0.82 (0.53–1.29)	1.50 (1.01–2.23)	1.52 (1.02–2.26)	0.0029	1.21 (1.06–1.37)

CKD	Model 1	1	1.03 (0.71–1.48)	1.68 (1.21–2.35)	2.02 (1.46–2.80)	< 0.0001	1.30 (1.18–1.44)
	Model 2	1	1.00 (0.69–1.45)	1.64 (1.17–2.29)	1.90 (1.37–2.63)	< 0.0001	1.28 (1.15–1.42)
	Model 3	1	0.97 (0.67–1.42)	1.59 (1.13–2.24)	1.72 (1.23–2.42)	< 0.0001	1.24 (1.11–1.38)
	Model 4	1	0.96 (0.66–1.41)	1.51 (1.07–2.15)	1.56 (1.10–2.20)	0.0013	1.20 (1.07–1.33)

*Note:* Data are presented as hazard ratios (95% confidence interval). Participants without increased urinary albumin excretion or CKD are defined as 0 and with increased urinary albumin excretion or CKD as 1. Model 1 is unadjusted. Model 2 is adjusted for age. Model 3 is adjusted for age, sex, physical activity, and SBP. Model 4 is adjusted for age, sex, physical activity, SBP, LDL-C, fasting glucose, fasting insulin, and γ-GGT.

## Data Availability

The datasets collected and/or analyzed during the current study will be available from the corresponding authors upon reasonable request.

## Nomenclature

CKD: Chronic kidney disease
VAI: Visceral adiposity index
UACR: Urine albumin-to-creatinine ratio
eGFR: Estimated glomerular filtration rate
γ-GGT: γ-glutamyltransferase
FPG: Fasting plasma glucose
LDL-C: Low-density lipoprotein cholesterol
SBP: Systolic blood pressure
WC: Waist circumference
BMI: Body mass index
TC: Total cholesterol
TG: Triglyceride
HDL-C: High-density lipoprotein cholesterol
CT: Computed tomography
SAT: Subcutaneous adipose tissue
IRB: Institutional Review Board
IPAQ: International Physical Activity Questionnaire
MET-h/week: Metabolic equivalent hours per week
ADA: American Diabetes Association
OGTT: Oral glucose tolerance test
HbA1c: Hemoglobin A1c
ASCVD: Arteriosclerotic cardiovascular disease
AST: Aspartate aminotransferase
ALT: Alanine aminotransferase
MDRD: Modification of Diet in Renal Disease
SD: Standard deviation
DBP: Diastolic blood pressure
CI: Confidence interval
HR: Hazard ratio
FFA: Free fatty acid
VLDL: Very-low-density lipoprotein
MRI: Magnetic resonance imaging
